# Repetitive transcranial magnetic stimulation may be superior to drug therapy in the treatment of Alzheimer's disease: A systematic review and Bayesian network meta‐analysis

**DOI:** 10.1111/cns.14228

**Published:** 2023-04-23

**Authors:** Naili Wei, Haoxin Liu, Wenrui Ye, Shengliang Xu, Changhao Lu, Anxiang Dai, Ting Hou, Xin Zeng, Jie Wu, Jian Chen

**Affiliations:** ^1^ Department of Neurosurgery The First Affiliated Hospital of Shantou University Medical College Shantou China; ^2^ Department of Neurosurgery, Xiangya Hospital Central South University Changsha China; ^3^ Department of Geriatrics The First Affiliated Hospital of Shantou University Medical College Shantou China; ^4^ Brain Function and Disease Laboratory Shantou University Medical College Shantou China

**Keywords:** Alzheimer's disease, Bayesian network meta‐analysis, cognitive function, drug therapy, repetitive transcranial magnetic stimulation

## Abstract

**Background:**

Repetitive transcranial magnetic stimulation (rTMS) is a noninvasive brain stimulation therapy that is primarily used to treat a variety of neuropsychiatric conditions. Recently, previous research reports stated that rTMS have the characteristics of neurorestorative in Alzheimer's disease (AD). However, the relevant clinical research evidence has not been fully summarized.

**Methods:**

This article performed a network meta‐analysis of individual participant data from eligible studies searched in PubMed, Embase, and the Cochrane Library from inception to March 31, 2022. The drug treatments involved were acetylcholinesterase inhibitors (AChEIs), *N*‐methyl‐d‐aspartate (NMDA), anti‐amyloid‐beta (Aβ), and some new targeted therapeutic drugs.

**Results:**

A total of 15, 548 individuals with AD disease in 57 randomized clinical trials (RCTs) were included in this meta‐analysis. The results indicated that the patients who received rTMS treatment (standard mean difference [SMD]: 0.65; 95% confidence interval [CI]: 0.22–1.07) had a better MMSE score than placebo. Treatment outcome analysis showed that, compared with multiple pharmacological interventions, rTMS acquired the greatest probability rank with the best cognitive improvement in MMSE score [the surface under the cumulative ranking curve (SUCRA) 93.3%] and ADAS‐cog score (SUCRA 86.7%). At the same time, rTMS treatment had the lowest rank in the adverse events (SUCRA 24.1%) except for the placebo group (SUCRA 19.1%).

**Conclusion:**

Compared with the current clinical drug treatment, rTMS demonstrated better cognitive function improvement and fewer adverse events in AD patients. Therefore, rTMS shows broad prospects in the treatment of Alzheimer's disease, and it is worth being widely popularized in clinic.

## Graphical abstract

Network of eligible comparisons in the analyses and the surface under the cumulativeranking curve (SUCRA) for cognitive function. Compared with the current clinical drug treatment, repetitivetranscranial magnetic stimulation (rTMS) demonstrated better cognitive function improvement and feweradverse events in Alzheimer's disease (AD) patients.

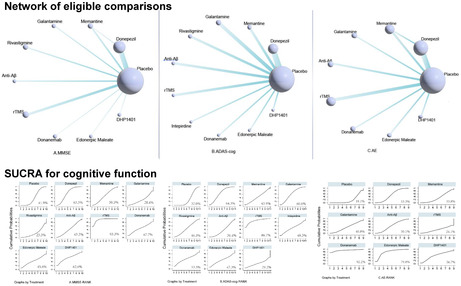

## 1 INTRODUCTION

Alzheimer's disease (AD) is a chronic, progressive, and serious nervous system disease, which can lead to memory loss and cognitive impairment.^1^ It accounts for half to 75% of all dementia cases, making it the most common cause.^2^ The 2015 World Alzheimer's Disease Report estimates that by 2030, the cost of treating dementia would rise from $818 billion in 2015 to $2 trillion.^3^, ^4^ In addition, the gradual dysfunction of AD patients brings huge cost to society and medical care.^5^ Currently, its standard treatment is pharmacotherapy, which includes acetylcholinesterase inhibitors (AChEIs), and *N*‐methyl‐d‐aspartate (NMDA), both of which have shown only little improvement in cognitive performance.^6^ Therefore, it is crucial to find alternate therapeutic methods for AD patients.

Transcranial magnetic stimulation (TMS) is a safe, economical, and noninvasive neuromodulation therapy.^7^ The core idea is to use Faraday's law of electromagnetic induction to generate a pulsed magnetic field that affects the central nervous system and to control the action potential of nerve cells through induced currents, thus affecting brain metabolism and neurophysiological processes.^8^ Recently, it has attracted more attention from neurologists due to its effects on improving brain activity and synaptic plasticity of neurons. According to the stimulation settings (such as stimulation frequency, stimulation of target sites, duration of stimulation, and inter stimulus interval), the specific regions of the cerebral cortex excitability can be increased or decreased by repeated TMS (rTMS).^8^, ^9^

With encouraging findings, several researchers have tried to treat AD with rTMS.^9^ However, rTMS therapy has not been included in the treatment guidelines for AD, nor has it been widely promoted in clinical practice.^10^ At the same time, the target and frequency of rTMS therapy are not standardized.^11^, ^12^ Therefore, it is necessary to comprehensively summarize the evidence to determine its clinical value, which will help promote the application of rTMS in AD.

Although previously published meta‐analysis studies revealed the effectiveness of rTMS, we were unable to conclude the advantages of rTMS over medication treatment for AD patients.^13^ Most of the existing studies were designed as blank controlled study trials to compare the outcomes of multiple drug therapy and placebo therapy or compare the effect of rTMS therapy and sham stimulation.^14^, ^15^ We conducted a network meta‐analysis of the results and adverse events in the current study to analyze the clinical advantages of rTMS treatment over pharmacotherapy. This network meta‐analysis will further promote the application of rTMS in the treatment of AD.

## 2 METHODOLOGY

### 2.1 Search strategy

Two investigators (HX‐L and NL‐W) independently searched the databases PubMed, Cochrane Library, and Embase for articles published up to March 31, 2022, without language restrictions. The following Emtree/MeSH words were used in the search: “Alzheimer's disease,” “randomized controlled trials,” “drug therapy,” “donepezil,” “memantine,” “rivastigmine,” “galantamine,” and “transcranial magnetic stimulation” integrated in the search algorithm with the relevant free terms tailored to each database. Disagreements about full‐text article eligibility were handled through discussion or arbitration with a third reviewer (JC). The search algorithms that used various databases are explained in the File S1.

### 2.2 Eligibility criteria

#### 2.2.1 Inclusion criteria

All randomized controlled trials available from inception until March 31, 2022, as a treatment for AD patients using recurrent transcranial magnetic stimulation or medication therapy, were considered for inclusion. Here are some details: (1) Participants solely had AD‐related cognitive impairment. (2) Both rTMS and drug therapy interventions were monotherapy rather than combination therapy. Drug therapy was recommended in the latest international guidelines for drugs and dosages, which included donepezil, memantine, galantamine, rivastigmine, and some newly developed drugs, such as anti‐Aβ, intepirdine, donanemab, edonerpic maleate, and DHP1401. (3) Placebo, no intervention, sham TMS, or other equivalent therapies served as the comparator.

#### 2.2.2 Exclusion criteria

Studies that matched the following criteria for exclusion were not included: (1) individuals having a diagnosis of cognitive impairment caused by an illness other than Alzheimer's disease; (2) irrelevant outcomes (not rTMS or drug therapy interventions as monotherapy); and (3) review articles, case reports, clinical protocols, conference abstracts, and nonhuman research.

### 2.3 Data extraction

Using a predetermined data extraction form, three authors (HL, WY, and SX) extracted the data from the included studies. Only the most recent and complete report was provided after duplicates were electronically eliminated. When the original data were not provided, we contacted the authors.

### 2.4 Quality assessment

Two independent investigators (HX‐L and NL‐W) accessed the quality of the included randomized controlled studies using Review Manager 5.4.1 according to the Cochrane risk‐of‐bias assessment tool. The following aspects were examined: (i) random sequence generation (selection bias), (ii) allocation concealment (selection bias), (iii) blinding of participants and personnel (performance bias), (iv) blinding of outcome assessment (detection bias), (v) incomplete outcome data (attrition bias), (vi) selective reporting (reporting bias), and (vii) other bias.

### 2.5 Primary and secondary outcomes

The treatment effect on overall cognitive function was the primary endpoint. The Mini‐Mental State Examination (MMSE) was applied to measure general cognitive function. Hence, pre‐post changes in MMSE scores were used to determine treatment effects for global cognition. An increase in the MMSE score after treatment indicated improved cognitive function.

The secondary outcome was the AD Assessment Scale‐Cognitive Subscale (ADAS‐cog) score, an internationally recognized and widely used test to assess cognitive function in mild‐to‐moderate Alzheimer's disease. Higher scores indicate lower performance on the neuropsychological test, which evaluates memory, language, praxis, and orientation as cognitive domains. This meta‐analysis also calculated the percentage of patients who dropped out of the study due to adverse events, which reflects patient acceptance of the treatment and can also be used to assess safety.

### 2.6 Statistical analyses

Both pairwise and network meta‐analyses were conducted at the same time. Using a random‐effects model, which served as the pooled effect sizes in traditional pairwise and network meta‐analysis, we calculated the standard mean difference (SMD) for continuous outcomes and odds ratios (ORs) for dichotomous outcomes along with the accompanying 95% confidence interval (CI). All statistical analyses were carried out using STATA, version 17.0, and a two‐sided *p*‐value < 0.05 was regarded as statistically significant. The frequentist model undertook this network meta‐analysis. The network graph was applied to illustrate all treatment comparisons for each outcome. The discrepancy between direct and indirect evidence in the loop was evaluated using a loop‐specific inconsistency technique. The consistency results were deemed insignificant when the 95% CIs of the inconsistency components included zero.

The surface under the cumulative ranking curve (SUCRA) displayed the average rating of each therapy for the best fictitious intervention. Indicators of a better rank of treatment benefit on cognitive impacts included a greater area under the curve.

In addition, for the nine included rTMS studies, an additional meta‐analysis was conducted. To explore the effect of rTMS on global cognition, a random‐effects meta‐analysis methodology was utilized due to the considerable diversity between studies. SMD and 95% CI were calculated and reported for two outcomes. Studies presenting two global cognitive outcome measures (MMSE, ADAS‐Cog).

The visually examining funnel plots generated by STATA, version 17.0, were applied to evaluate publication bias in studies that contributed to primary outcomes and adverse events.

## 3 RESULTS

### 3.1 Literature search results

**FIGURE 1:**
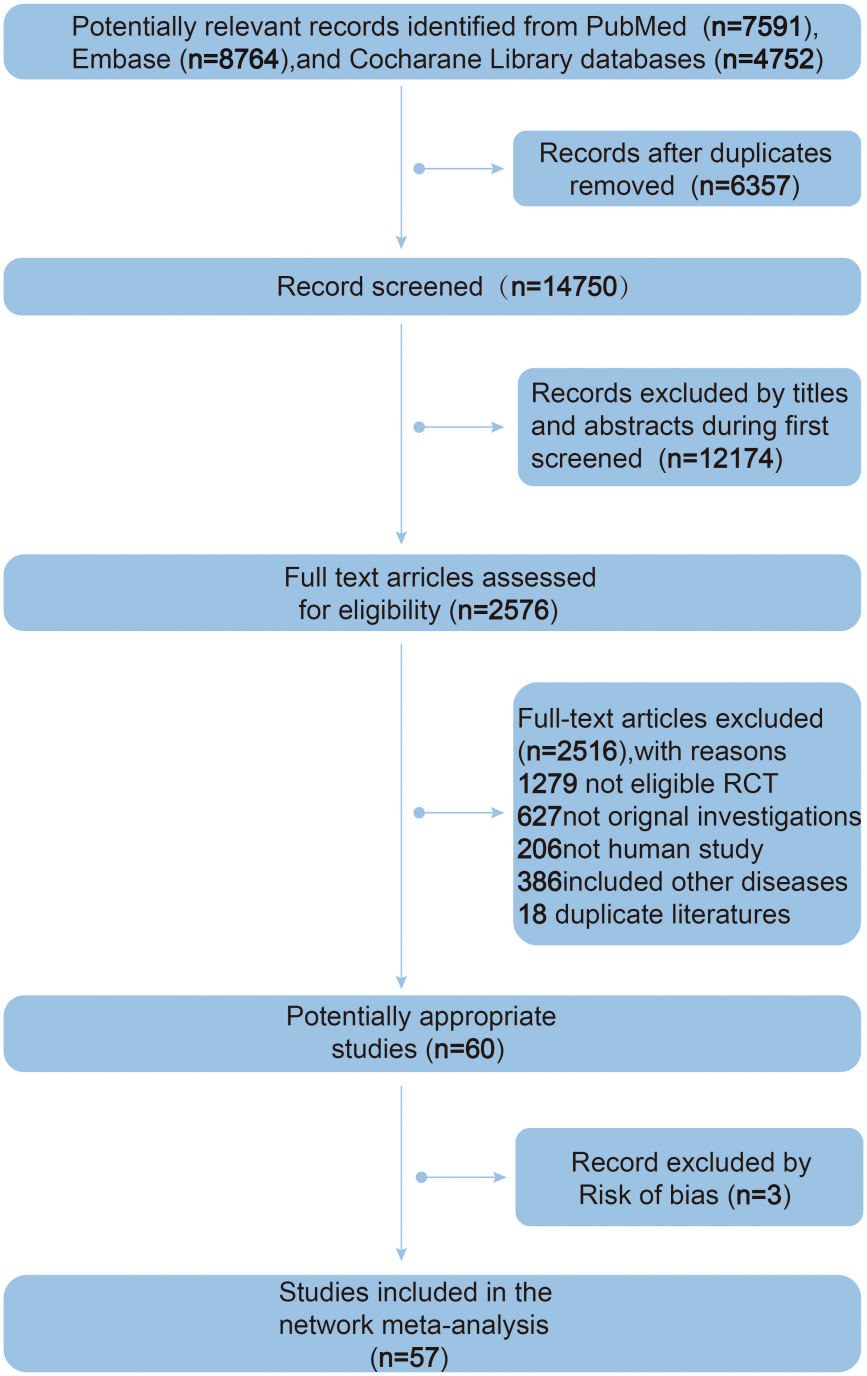
Flow chart showing literature selection.

In the initial examination, a total of 21,107 relevant papers were retrieved using the study's search approach. The titles and abstracts for 14,750 citations were obtained after Endnote X9 software eliminated 6357 duplicate citations. Then, we screened titles and abstracts to exclude 12,174. Subsequently, the eligibility of 2576 pertinent full‐text articles was evaluated. 2516 citations were dropped, comprising 1279 not eligible RCT, 627 not original investigations, 206, not a human study, 386 included other types of disease, and 18 duplicate kinds of literature. Three studies were excluded by the risk of bias. Ultimately, 57 studies were included in this network meta‐analysis. The search flow diagram is shown in Figure 1.

### 3.2 General characteristics

Fifty‐seven studies, including 15,548 patients, were eligible for analysis. Between 1998 and 2022, each of these investigations was published in an English‐language publication. Interventions in these studies were categorized into drug therapy and rTMS. The relevant characteristics were extracted, including treatment, sample size, age, gender, baseline MMSE score, criteria for AD, and duration.

For the subjects in nine rTMS trials, eight studies stimulated the left, right, or bilateral dorsolateral prefrontal cortex (DLPFC). Broca's and Wernicke's areas, the right and left DLPFC, the right and left parietal somatosensory association cortex, and the right and left Wernicke's area were all included in one study that focused on six brain regions that represented the location of the main centers involved in the manifestation of the clinical symptoms of AD (pSAC). According to the electroencephalogram 10–20 system, the remaining study activated the parietal P3/P4 and posterior temporal T5/T6. High‐frequency pulses (10–20 Hz) were used as a treatment parameter in all nine rTMS investigations. And in one study, they applied high‐frequency (10 Hz) stimulation to the left DLPFC, followed by low‐frequency (1 Hz) stimulation to the right DLPFC. Of the 48 drug treatment studies included, 18 were AChEIs (such as donepezil, galantamine, and rivastigmine), nine were NMDA (such as memantine), four were anti‐amyloid‐beta (Aβ; such as semagacestat, verubecestat, tarenflurbil, and solanezumab), and four were new targeted therapeutic drugs (such as intepirdine, donanemab, edonerpic maleate, and DHP1401).

**FIGURE 2:**
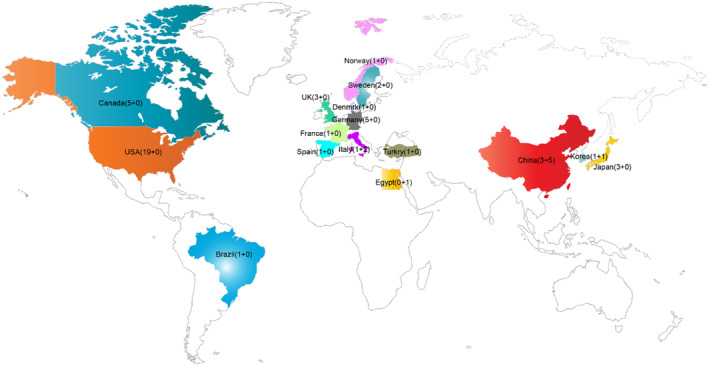
Map depicting studies eligible for systematic review. Note: Country, the number of included studies (Drug treatment + rTMS).

Table 1 displays the particular aspects of the included studies as well as the patient information. As shown in Figure 2, these RCTs were conducted in 16 countries. Most drug studies (*n* = 19 [39.6%]) were conducted in the United States, while most rTMS studies (*n* = 5 [55.6%]) were conducted in China.

**TABLE 1:** Characteristics of studies used for analysis.

			Age	Gender	Baseline MMSE		Duration
Study	Treatment	*N*	Mean (SD)	(% female)	Mean (SD)	Criteria	(weeks)
Andersen et al., 2012	Donepezil	90	80.8 (6.8)	67	23.2 (4.2)	NINCDS‐ADRDA	12
	Placebo	90	80.8 (7.3)	54	23.1 (4.1)		
Black et al., 2007	Donepezil	176	78 (8.04)	72.7	7.5 (3.23)	NINCDS‐ADRDA	24
	Placebo	167	78 (8.2)	67.7	7.5 (3.51)		
Cristina et al., 2013	Donepezil	7	73 (5.54)	85.7	21.86 (4.45)	NINCDS‐ADRDA	12
	Placebo	7	76.6 (7.9)	71.4	23.57 (2.22)		
Feldman et al., 2001	Donepezil	144	73.3 (10)	61.1	11.72 (3)	NINCDS‐ADRDA	24
	Placebo	146	74 (11)	61	11.97 (3.25)		
Frolich et al., 2010	Donepezil	158	73.9 (6.48)	65.8	20.6 (3.6)	NINCDS‐ADRDA	12
	Placebo	163	73.5 (6.42)	55.2	20.7 (3.7)		
Gault et al., 2015	Donepezil	68	72.4 (8.42)	45.6	19.6 (3.82)	NINCDS‐ADRDA	12
	Placebo	68	73.6 (8.23)	61.8	19.7 (3.95)		
Haig et al., 2014	Donepezil	60	70.5 (8.31)	60	18.1 (4.1)	NINCDS‐ADRDA	12
	Placebo	63	70.3 (7.84)	61.9	18.2 (3.9)		
Homma et al., 2000	Donepezil	116	70.1 (7.6)	68	17.8 (3.9)	DSM‐IV	24
	Placebo	112	69.4 (8.8)	66	16.6 (3.9)		
Howard et al., 2007	Donepezil	128	84.9 (7.3)	82	8.1 (5.9)	NINCDS‐ADRDA	12
	Placebo	131	84.4 (8.2)	87	8.2 (6.8)		
Johannsen et al., 2006	Donepezil	99	74.1 (7.6)	59.6	18.8 (4.8)	NINCDS‐ADRDA	12
	Placebo	103	71.4 (9.3)	63.1	18.5 (4.8)		
Mazza et al., 2006	Donepezil	25	64.5 (6.0)	48	18.55 (3.47)	DSM‐IV	24
	Placebo	26	69.8 (3.0)	61	18.80 (3.63)		
Moraes et al., 2008	Donepezil	11	76.8 (6.2)	72.7	19 (3.6)	NINCDS‐ADRDA	12
	Placebo	12	72.6 (11)	58.3	17.2 (7.8)	DSM‐IVR	
Peng et al., 2005	Donepezil	46	72.6 (6.8)	54.3	17.8 (2.3)	NINCDS‐ADRDA	12
	Placebo	43	71.8 (8.2)	55.8	18.2 (2.7)		
Petersen et al., 2005	Donepezil	253	73.1 (7.1)	44	27.25 (1.8)	NINCDS‐ADRDA	144
	Placebo	259	72.9 (7.6)	47	27.35 (1.8)		
Rogers et al., 1998	Donepezil	157	73.8 (8.4)	69	19.4 (4.9)	DSM‐III‐R	12
	Placebo	158	73.4 (8.2)	61	19.4 (5.0)	NINCDS‐ADRDA	
Winblad et al., 2006	Donepezil	128	84.5 (6.0)	79	6.0 (3.0)	DSM‐IV	24
	Placebo	120	85.3 (5.9)	74	6.2 (3.0)	NINCDS‐ADRDA	
Cao et al., 2020	Memantine	154	74.4 (9.4)	31.8	8.7 (2.1)	NA	12
	Donepezil	156	74.5 (9.5)	30.8	8.8 (2.7)		
Castelar et al., 2019	Donepezil	12	79.3 (7.4)	66.7	24.3 (3.5)	NINCDS‐ADRDA	6
	Placebo	11	81.7 (3.8)	63.6	25.4 (1.7)		
Ashford et al., 2011	Memantine	7	76.5 (9.6)	14	19.9 (4.8)	NINCDS‐ADRDA	24
	Placebo	6	75.4 (6.3)	67	21.8 (3.1)		
Dysken et al., 2014	Memantine	155	78.8 (7.2)	4	20.8 (3.8)	NA	48
	Placebo	152	79.4 (7.0)	2	20.8 (3.8)		
Fox et al., 2012	Memantine	72	84.9 (6.7)	34.7	7.3 (6.2)	NINCDS‐ADRDA	12
	Placebo	77	84.4 (6.6)	75.3	7.3 (6.4)		
Nakamura et al., 2014	Memantine	318	74 (8.9)	67	10.1 (2.94)	NINCDS‐ADRDA	24
	Placebo	315	74.5 (8.6)	67	9.9 (2.96)		
Peskind et al., 2006	Memantine	201	78 (7.3)	60.2	17.4 (3.7)	NINCDS‐ADRDA	24
	Placebo	202	77 (8.2)	57.4	17.2 (3.4)		
Reisberg et al., 2003	Memantine	126	75.5 (8.16)	72.2	7.8 (3.76)	NINCDS‐ADRDA	28
	Placebo	126	75.8 (7.28)	65.5	8.1 (3.6)	DSM‐IV	
Reisberg et al., 2006	Memantine	95	75.5 (8.24)	64	7.78 (3.75)	NINCDS‐ADRDA	28
	Placebo	80	75.6 (7.15)	72	8.05 (3.65)		
Wang et al., 2013	Memantine	11	65.7 (12.5)	63.3	14.1 (4.6)	NINCDS‐ADRDA	24
	Placebo	11	64.7 (11.5)	63.3	10.1 (6.1)	DSM‐IV	
Blautzik et al., 2016	Galantamine	14	NA	NA	20.33 (1.89)	NINCDS‐ADRDA	48
	Placebo	11	NA	NA	22 (3.46)		
Hager et al., 2014	Galantamine	1024	73.0 (8.9)	65.5	19.0 (4.12)	NINCDS‐ADRDA	104
	Placebo	1021	73.0 (8.7)	64.1	19.0 (4.04)		
Likitjaroen et al., 2011	Galantamine	14	73.5 (7.2)	57.1	22.1 (2.4)	NINCDS‐ADRDA	48
	Placebo	11	76.4 (7.9)	63.6	23.0 (3.5)		
Raskind et al., 2000	Galantamine	212	75.9 (7.3)	65.57	19.5 (4.4)	NINCDS‐ADRDA	24
	Placebo	213	75.3 (8.8)	61.5	19.2 (4.4)		
Rockwood et al., 2001	Galantamine	261	75.2 (7.3)	56.7	19.7 (3.88)	NINCDS‐ADRDA	12
	Placebo	125	74.6 (7.6)	53.6	19.6 (3.58)		
Rockwood et al., 2006	Galantamine	64	77.0 (8.0)	64	20.8 (3.3)	NINCDS‐ADRDA	16
	Placebo	66	78.0 (8.0)	62	19.9 (4.2)		
Tariot et al., 2000	Galantamine	140	76 (7.1)	64.3	18 (3.6)	NINCDS‐ADRDA	20
	Placebo	286	77.1 (8.5)	62.2	17.7 (3.4)		
Wilcock et al., 2000	Galantamine	220	71.9 (8.3)	63.2	19.5 (3.4)	NINCDS‐ADRDA	24
	Placebo	215	72.7 (7.6)	61.4	19.3 (3.5)		
Wilkinson et al., 2001	Galantamine	88	72.7 (8.4)	56	18.8 (2.8)	NINCDS‐ADRDA	12
	Placebo	87	74.2 (8.4)	59	18.7 (2.8)	DSM‐III‐R	
Feldman et al., 2007	Rivastigmine	227	71.4 (7.9)	60	18.3 (4.5)	NINCDS‐ADRDA	26
	Placebo	222	71.7 (8.7)	60	18.7 (4.6)		
Karaman et al., 2004	Rivastigmine	24	74.11 (4.3)	54.17	11.40 (1.0)	DSM‐IV	52
	Placebo	20	73.40 (4.0)	55	13.20 (0.9)	NINCDS‐ADRDA	
Nakamura et al., 2011	Rivastigmine	282	74.3 (7.5)	68.8	16.8 (2.9)	DSM‐IV	24
	Placebo	286	74.5 (7.4)	68.2	16.6 (2.9)	NINCDS‐ADRDA	
Rosler et al., 1999	Rivastigmine	243	72 (11.75)	59	NA	NINCDS‐ADRDA	26
	Placebo	239	72 (11.75)	59		DSM‐IV	
Winblad et al., 2007	Rivastigmine	291	73.6 (7.9)	68	16.6 (3.1)	NINCDS‐ADRDA	24
	Placebo	302	73.9 (7.3)	66.6	16.4 (3.0)	DSM‐IV	
Doody et al., 2013	Semagacestat	527	73.3 (8.5)	50	20.7 (3.5)	NINCDS‐ADRDA	76
	Placebo	501	73.3 (8.1)	45	20.9 (3.6)		
Egan et al., 2018	Verubecestat	652	71.8 (7.6)	58.1	20.2 (3.3)	NA	78
	Placebo	653	72.4 (7,6)	54.2	20.3 (3.3)		
Green et al., 2009	Tarenflurbil	840	74.6 (8.5)	49.4	23.3 (2.0)	NINCDS‐ADRDA	72
	Placebo	809	74.7 (8.4)	52.5	23.3 (2.0)	DSM‐IV	
Honig et al., 2018	Solanezumab	1057	72.2 (7.8)	56.8	22.8 (2.8)	NINCDS‐ADRDA	76
	Placebo	1072	73.3 (8.0)	58.9	22.6 (2.9)		
Ahmed et al., 2012	rTMS	15	65.9 (5.9)	66.7	14.7 (3.7)	NINCDS‐ADRDA	12
	Sham coil	15	68.3 (4.9)	66.7	13.9 (3.9)		
Bagattini et al., 2020	rTMS	27	73.56 (4.91)	37.3	23.67 (3)	NA	12
	Sham coil	23	73.35 (1.09)	47.8	22.77 (0.6)		
Cotelli et al., 2011	rTMS	5	71.2 (6.1)	NA	16.2 (2.7)	NINCDS‐ADRDA	12
	Sham coil	5	74.4 (3.8)		16 (2.0)		
Jia et al., 2021	rTMS	35	71.41 (8.85)	71.43	15.71 (5.6)	NINCDS‐ADRDA	2
	Sham coil	34	73.41 (7.73)	67.65	15.62 (6.49)	DSM‐V	
Lee et al., 2016	rTMS	18	72.1 (7.6)	55.6	22.4 (2.9)	NINCDS‐ADRDA	6
	Sham coil	8	70.3 (4.8)	62.5	22.8 (2.5)	DSM‐IV	
Li et al., 2021	rTMS	37	65.97 (8.47)	45.9	16.13 (4.27)	DSM‐V	6
	Sham coil	38	64.58 (7.88)	36.8	15.97 (4.12)		
Wu et al., 2015	rTMS	26	71.4 (4.9)	61.5	15.3 (3.1)	NINCDS‐ADRDA	4
	Sham coil	26	71.9 (4.8)	57.7	15.2 (3.1)		
Zhao et al., 2017	rTMS	17	69.3 (5.8)	58.8	22.2 (2.8)	NA	6
	Sham coil	13	71.4 (5.2)	54.8	22.8 (2.3)		
Zhou et al., 2021	rTMS	33	70 (3)	66.7	19 (0.7)	NINCDS‐ADRDA	4
	Sham coil	32	74 (3.9)	68.8	19.1 (0.57)		
Lang et al., 2021	Intepirdine	643	72.7 (7.68)	60	18.5 (3.7)	NINCDS‐ADRDA	24
	Placebo	633	72.5 (7.55)	62.2	18.5 (3.63)		
Mintun et al., 2021	Donanemab	131	75 (5.6)	51.9	23.6 (3.1)	NINCDS‐ADRDA	76
	Placebo	126	75.4 (5.4)	51.6	23.7 (2.9)		
Schneider et al., 2019	Edonerpic Maleate	159	71.9 (8.2)	50.9	18.2 (3.8)	NINCDS‐ADRDA	52
	Placebo	156	71.8 (7.5)	57.1	18.2 (3.8)	DSM‐IV	
Shim et al., 2022	DHP1401	58	73.78 (7.18)	58.6	20.34 (3.18)	NINCDS‐ADRDA	24
	Placebo	64	73.56 (7.77)	67.2	19.77 (2.9)		

Abbreviations: DSM‐III‐R, Diagnostic and Statistical Manual of Mental Disorders third edition, revision; DSM‐IV, Diagnostic and Statistical Manual of Mental Disorders, Fourth Edition; DSM‐V, Diagnostic and Statistical Manual of Mental Disorders, Fifth Edition; MMSE, Mini‐mental State Examination; NA, not available; NINCDS‐ADRDA, The National Institute of Neurological and Communicative Disorders Association and Stroke‐AD and Related Disorders Association.

### 3.3 Network plots of eligible comparisons

**FIGURE 3:**
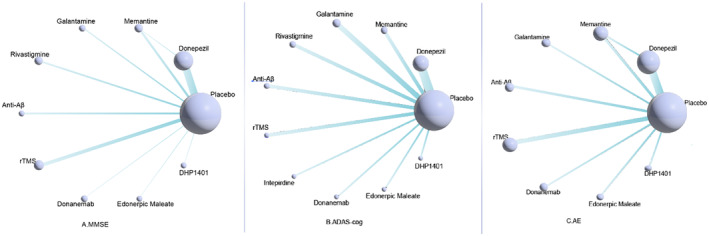
Network of eligible comparisons for all treatments included in the analyses. Note: (A) MMSE：Mini‐Mental State Examination, (B) ADAS‐cog：Alzheimer´s Disease Assessment Scale‐Cognitive, (C) AE：Adverse Events.

Figure 3 suggests that placebo was the most common control across all studies, with no studies directly comparing drug treatment with rTMS. The mesh diagram of the primary outcome indicators (MMSE) is reflected in Figure 3A. The included trials yielded 10 nodes contributing to 10 pairs of comparisons, with donepezil and memantine being directly compared. Comparisons of secondary measures (ADAS‐cog and AE) are shown in Figure 3B,C, respectively. The included trials yielded 11 nodes, contributing to 10 pairs of comparisons. Meanwhile, the included trials yielded nine nodes for adverse events, facilitating nine pairs of comparisons.

### 3.4 Primary outcomes

Data regarding the effects of drug therapy or rTMS on primary outcomes (MMSE scores) were available from 45 trials with 13,185 patients. There were eight studies with 346 participants comparing rTMS treatment to placebo. 12,839 people were involved in 36 drug treatment versus placebo investigations and one drug comparison study, which compared donepezil and memantine.

For the pairwise meta‐analysis, rTMS was more significantly effective than the placebo groups (SMD: 0.65; 95% CI: 0.22–1.07), anti‐Aβ (SMD: 0.64; 95% CI: 0.00–1.28), galantamine (SMD: 0.83; 95% CI: 0.04–1.62), and rivastigmine (SMD: 0.86; 95% CI: 0.20–1.53; Table 2A; *p* < 0.05). Only rTMS significantly increased MMSE scores compared with the placebo groups.

**TABLE 2:** Traditional pairwise meta‐analysis on efficiency and safety.

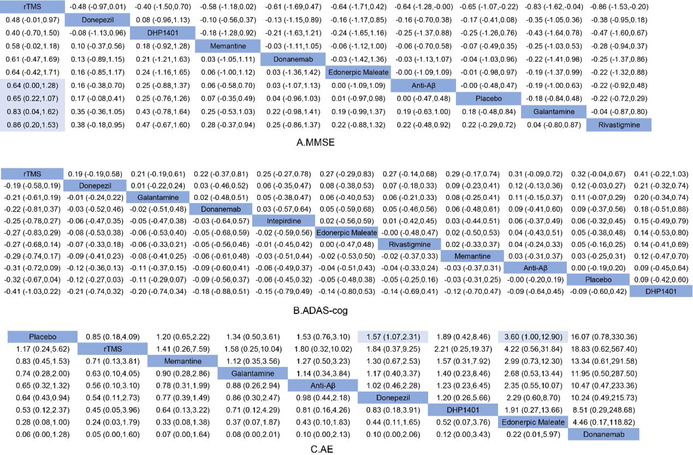	

Abbreviations: A. MMSE, Mini‐mental State Examination, (SMD [95% CI]). B. ADAS‐cog, Alzheimer's Disease Assessment Scale‐Cognitive section, (SMD [95% CI]); C. AE, Adverse Events (OR [95% CI]).

**FIGURE 4:**
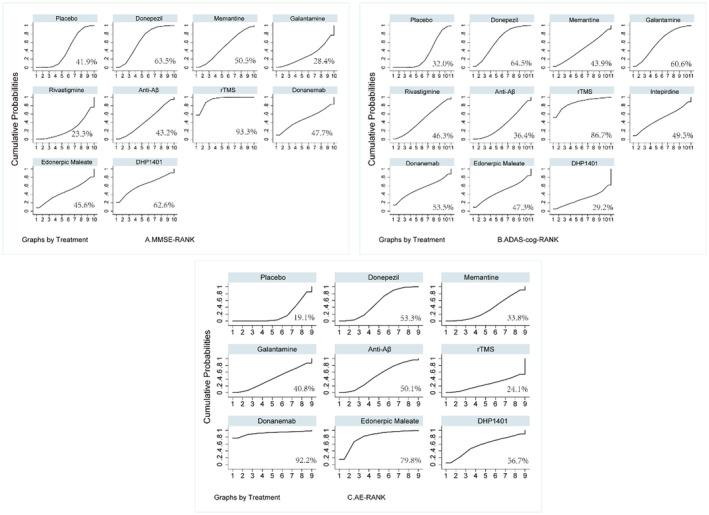
SUCRA for cognitive function based on MMSE(A), ADAS‐cog scale (B), and AE (C). Note: SUCRA, the surface under the cumulative ranking curve; MMSE, Mini‐mental State Examination; ADAS‐cog, Alzheimer’s Disease Assessment Scale‐cognition subscale; AE, Adverse Events.

Ranking the effects of all interventions by SUCRA probability, we found that rTMS had the highest likelihood (SUCRA 93.3%) of being the best intervention to improve MMSE score, followed by donepezil (63.5%) and DHP1401 (62.6%), and the least effective was Rivastigmine (23.3%; Figure 4A).

The pooled meta‐analysis of eight trials that examined the effects of rTMS versus sham on patients with AD found that the former significantly enhanced patients' global cognition (as judged by the MMSE; SMD = 0.70; 95% CI = 0.07, 1.34; *p* = 0.03; *I* ^2^ = 85.9%, File S2A).

### 3.5 Secondary outcome

ADAS‐cog scores analysis, which is one of the secondary outcomes, is shown in Table 2B. The network of ADAS‐cog included 37 studies involving 14,227 patients with 11 interventions. The consistency model was used to compare these various treatments after Node‐splitting analysis. Compared with other pharmacological interventions, rTMS showed good improvement in cognitive function, although there was no statistical difference.

The effects of all interventions were ranked with SUCRA probabilities (Figure 4B). Similar to MMSE, rTMS had the highest chance of being the best intervention on ADAS‐cog (SUCRA 86.7%), followed by donepezil (SUCRA 64.5%) and galantamine (SUCRA 60.6%). DP1401 came in last (SUCRA 29.2%). It can be seen that for the ADAS‐cog scale, rTMS likewise showed a better advantage over pharmacotherapy.

In addition, the result of adverse events was available from 26 trials with 8719 patients. Adverse events associated with donepezil (OR: 1.57; 95% CI: 1.07–2.31; Table 2C) and edonerpic maleate (OR: 3.60; 95% CI: 1.00–12.90) were substantially more common than those associated with placebo (*p* < 0.05). The adverse events of all interventions were ranked with SUCRA probabilities (Figure 4C). The placebo group had the lowest probability (SUCRA 19.1%) for adverse events with certain common sense. Meanwhile, the rTMS group ranked in the second position (SUCRA 24.1%), suggesting that rTMS is the safest among all the interventions.

The pooled meta‐analysis of four trials that looked at the impact of rTMS on global cognition in AD in comparison with sham treatment found no appreciable improvement in global cognition (as measured by ADAS‐cog; SMD = −0.32; 95% CI = −0.68, 0.04; *p* = 0.07; *I* ^2^ = 26.7%, File S2B).

Similar to many medications, the main adverse effects of donepezil were diarrhea, insomnia, nausea, vomiting, and anxiety, which made patients intolerable and refuse to continue medication. In contrast, adverse events were less in the rTMS group, and patients generally experienced tolerable mild headaches. Therefore, rTMS is currently the safer treatment option.

### 3.6 Quality assessment

**FIGURE 5:**
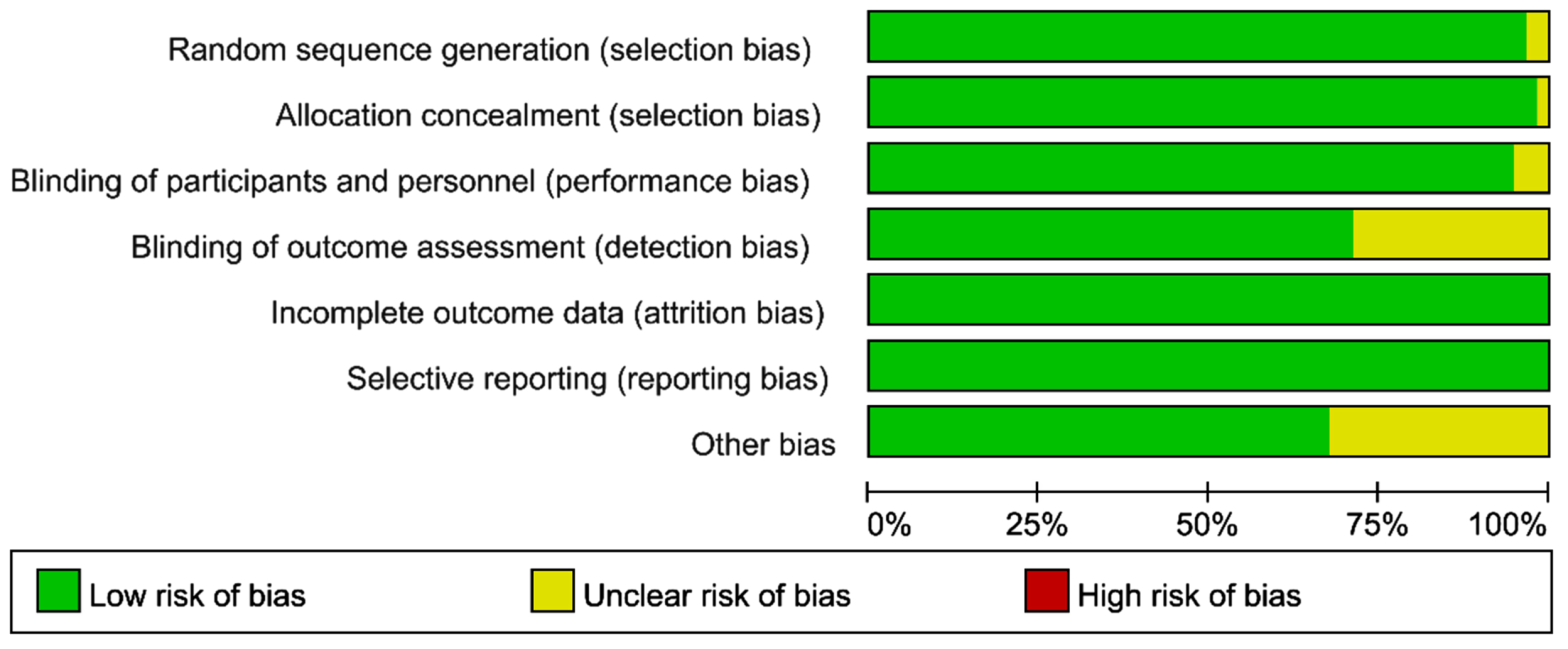
Risk of bias graph.

Two (3.5%) randomized clinical trials showed a low risk of bias due to inadequate sequence generation. Regarding allocation concealment, one (1.7%) trial had a low risk since they used an opaque envelope or central randomization system. Three (5.3%) trials were not blinded to participants and personnel, while 16 (28.1%) were not blinded to outcome assessment. In all randomized clinical studies, the likelihood of selective reporting bias and incomplete outcome data was minimal. In all included trials, other biases were unclear. Therefore, the overall quality of the incorporated articles is very high. Figure 5 and File S3, respectively, show the risk‐of‐bias graph and risk‐of‐bias summary for selected studies.

### 3.7 Small sample effect detection

Comparison‐adjusted funnel plots were created for the included studies (File S4). Points of the same color in the funnel diagram symbolized pairwise comparisons in the original study, and points of different colors represented various pairwise direct comparisons. Symmetrical funnel plots (File S4) indicated possible small sample effects or little chance of publication bias for the three outcome measures between treatment measures.

## 4 DISCUSSION

As far as we all know, this is the first study to compare the efficacy and treatment‐related adverse reactions of rTMS with pharmacological treatments for AD through a network meta‐analysis. The results of the present network meta‐analysis were encouraging, with rTMS superior to pharmacological treatment in terms of therapeutic efficacy and improvement in cognitive function. When comparing MMSE scale with ADAS‐cog scale, the same conclusion was obtained. Furthermore, regarding adverse treatment reactions, the adverse reaction of rTMS is obviously less than that of drug treatment, which was close to that of placebo group. Our results demonstrate the advantages and prospect of rTMS treatment compared with traditional drug therapy.

In the brains of AD patients, cholinergic neurons and circuits show the highest degrees of degradation, and cholinergic drugs have been used to relieve symptoms.^16^ Acetylcholinesterase is inhibited by donepezil, rivastigmine, and galantamine, which increases the amount of available acetylcholine in the brain.^17^ Memantine is a noncompetitive NMDA receptor antagonist with low affinity, which can prevent glutamate‐induced neurotoxicity.^18^ J et al. found that after 6 months of treatment with donepezil, galantamine, or rivastigmine, the cognitive function of patients at the center of the ADAS‐Cog 70‐point scales improved by a −2.7 points (95% CI: −3.0, −2.3) on average.^19^ The current network meta‐analysis included the abovementioned classic drugs and targeted therapeutics developed recently.^20^ Although not all commercially available drugs were included in this study, classical and clinically used drugs were included and are fully representative of the current state of drug therapy. This network meta‐analysis suggested that donepezil could improve cognitive function of AD patients, which was similar to previous literature reports.^14^ Because the outcomes of recently discovered targeted treatments were inconsistent, it needs a longer time and larger scale of studies to examine their efficacy and safety in AD.^21^ A systematic analysis focused on the effects of rTMS in Alzheimer's disease patients found that rTMS may have a favorable effect on cognitive function in AD patients with good treatment results,^22^ which is consistent with our results. Transcranial magnetic stimulation can also affect the cortical and subcortical regions around the stimulation site remotely.^23^ It has also been shown to cause aftereffects by generating long‐term potential or inhibiting synaptic activity.^24^ Yanli et al.^25^ discovered that after 6 weeks of treatment, the average ADAS‐cog score was improved by 3.76 points compared to 0.47 in the control group and 3.52 points after 4.5 months of therapy compared with a worsening of 0.38 in the control group (*p* = 0.04 and *p* = 0.05, respectively). Most of the studies we included had a follow‐up time of 1–3 months. In future RCT studies, it seems appropriate to extend the follow‐up time to observe the best treatment effect.^26^, ^27^, ^28^ This sustained effect of rTMS is needed for AD patients. It seems to be equivalent to taking a long‐acting drug, which will benefit more elderly patients in future clinical applications.

In this study, the adverse events of drug therapy and transcranial magnetic stimulation were counted. Overall, rTMS was significantly superior to drug therapy. The previous study showed that the overall adverse reaction rate of rTMS was close to 5%, and the most common adverse reactions were mild headache and fatigue,^29^ and the most serious adverse reaction was eye twitching,^30^ accounting for 18.3%–26% of adverse drug reactions rate reported in previous literature.^31^ Whereas approximately 15% of people who received drug treatment withdrew from the study because of adverse events, the incidence of adverse reactions is even higher, and 50%–100% of adverse drug reactions were reported in previous literature.^32^ Among drug treatments, donanemab has the most increased adverse effects.^21^ The most serious adverse reactions reported by donanemab were nausea and infusion‐related reactions.^21^ In 2012, 1449 nursing home patients with severe cognitive impairment were examined as part of the Services and Health for Elderly in Long Term Care (SHELTER) research. According to the survey results, more than 45% of patients used inappropriate drugs. Due to undesirable pharmacological effects or a perceived lack of effectiveness, only half of the patients were assigned to continue donepezil for the duration of the research.^33^ For elderly patients, the response to many drugs was not ideal due to tolerance problems and decreased metabolism of liver and kidney function. Severe cognitive impairment can also lead to irregular medication taking and poor compliance.^34^ Therefore, rTMS also seems superior in terms of adverse effects.

Although the present study revealed that transcranial magnetic stimulation had clinical advantages over drug treatment, the conclusion might not be immutable. Recently, it has been reported that donanemab has a potential therapeutic effect on AD.^21^ With the continuous investment in a drug research and development, the emergence of new targeted drugs may change the result of this network meta‐analysis. The purpose of this study was to prove that TMS is a therapeutic strategy superior to drug therapy, which should be widely developed and applied. However, it still needs a larger sample size and long‐term follow‐up study to determine the best technology and check the long‐term effectiveness of rTMS in AD, which is one of the limitations of our study. In addition, according to previous reports, stimulation frequency and target area were critical to determining the optimal neuromodulation effect.^8^ Through further exploration, we found that of the nine rTMS studies we included, their stimulus parameters have certain commonalities. For example, most researchers unanimously chose the left DLPFC as the target of stimulation, and the coil location was checked using a stereotaxic neuronavigation device throughout the whole stimulation session.^27^ The DLPFC is a crucial network integration hub, mediating organizational and executive processes that may function across a wide range of tasks. Pathological alterations and dysfunction of the DLPFC are hallmark features of AD from its early stage.^35^, ^36^ Sole Padulles and his colleagues^37^ first proved in 2006 that high‐frequency rTMS applied to the left prefrontal cortex enhanced face‐name association memory of MCI patients. Since then, the DLPFC has been the target of the majority of rTMS therapies at various stages of AD pathology,^38^ which we also explored fully in this study. And in terms of stimulation frequency, all researchers chose high‐frequency therapy. To counteract the loss of brain function, high‐frequency pulses (10–20 Hz) appeared to improve cortical excitability in the majority of patients by increasing the excitability and activity of surviving cells.^39^ With the further development of rTMS in future clinical practice, we believe that the best treatment strategy may be exploited, and the overall therapeutic benefit of rTMS will be more significant.

## 5 CONCLUSION

This network meta‐analysis suggested that rTMS was more effective than current pharmacological treatment in improving global cognition, with significantly fewer adverse effects. Therefore, rTMS shows broad prospects in treating Alzheimer's disease and deserves to be widely promoted in clinical practice.

## AUTHOR CONTRIBUTIONS

The study's original concept was created by HL and NW, along with the study's scope, methodological approach, computer code, and meta‐analysis. The search strings were created, the search was carried out, the results were exported, and duplicate records were eliminated by HL, WY, and SX. The systematic review abstracts and texts were reviewed by HL, JW, CL, XZ, AD, and TH. They also retrieved pertinent data from the systematic review articles and assessed the papers' quality. The original draft of the paper was written by HL, NW, and JW, and it was critically revised by all of the writers. Study guarantors are NW, JW and JC. Senior and corresponding authors NW and JC each made an equal contribution to this work. All authors had complete access to all the study's data, and the corresponding authors were ultimately in charge of deciding whether to submit the work for publication. The corresponding author certifies that all mentioned authors satisfy the requirements for authorship and that no other writers who satisfy the requirements have been excluded.

## FUNDING INFORMATION

This work was supported by grants from the National Nature Science Foundation (82001447), the Natural Science Foundation of Guangdong Province(2022A1515010687), special funds for Guangdong Special Funds for Science and Technology, (STKJ2021075 and STKJ2021077), Guangdong Basic and Applied Basic Research Foundation (22202104030000234), and 2020 Li Ka Shing Foundation Cross‐Disciplinary Research Grant (2020LKSFG11C). The study's design, data collection, analysis, interpretation, report writing, and the choice to submit the article for publication were all made independently of the funders.

## CONFLICT OF INTEREST STATEMENT

None declared.

## Supporting information


Data S1:

Data S2:

## Data Availability

The data that support the findings of this study are available from the corresponding author upon reasonable request.
