# Supplementary material for: Repetitive transcranial magnetic stimulation may be superior to drug therapy in the treatment of Alzheimer's disease: A systematic review and Bayesian network meta‐analysis
Source: CNS Neurosci Ther. 2023 Apr 23;29(10):2912–24. doi: 10.1111/cns.14228 (PMC10493651; doi:10.1111/cns.14228)
Supplement: Supplementary file 1 — Data S1: [file CNS-29-2912-s001.doc]

**Search strategy for Pubmed**

#1 "Alzheimer Disease"[Mesh]

#2 (((Alzheimer Disease [Title/Abstract]) OR Alzheimer's Disease [Title/Abstract]) OR Alzheimer Dementia [Title/Abstract]) OR Alzheimer's Dementia [Title/Abstract]

#3 #1 OR #2

#4 "Transcranial Magnetic Stimulation"[Mesh]

#5 (Transcranial Magnetic Stimulation [Title/Abstract]) OR Magnetic Stimulation, Transcranial [Title/Abstract]

#6 #4 OR #5

#7 "Drug Therapy"[Mesh]

#8 Therapy, Drug[Title/Abstract] OR Drug Therapies[Title/Abstract] OR Therapies, Drug[Title/Abstract] OR Chemotherapy[Title/Abstract] OR Chemotherapies[Title/Abstract] OR Pharmacotherapy[Title/Abstract] OR Pharmacotherapies[Title/Abstract] OR Inhibitors, Cholinesterase[Title/Abstract] OR Anticholinesterase Drugs[Title/Abstract] OR Drugs, Anticholinesterase[Title/Abstract] OR Anticholinesterases[Title/Abstract] OR Anticholinesterase Agents[Title/Abstract] OR Agents, Anticholinesterase[Title/Abstract] OR Anti-Cholinesterases[Title/Abstract] OR Anti Cholinesterases[Title/Abstract] OR Cholinesterase Inhibitors, Irreversible[Title/Abstract] OR Inhibitors, Irreversible Cholinesterase[Title/Abstract] OR Irreversible Cholinesterase Inhibitors[Title/Abstract] OR Cholinesterase Inhibitors, Reversible[Title/Abstract] OR Inhibitors, Reversible Cholinesterase[Title/Abstract] OR Reversible Cholinesterase Inhibitors[Title/Abstract] OR Acetylcholinesterase Inhibitors[Title/Abstract] OR Inhibitors, Acetylcholinesterase[Title/Abstract] OR Memantine[Title/Abstract] OR donepezil[Title/Abstract] OR rivastigmine[Title/Abstract] OR galantamine[Title/Abstract].

#9 #7 OR #8

#10 #6 OR #9

#11 "randomized controlled trial"[pt] OR "controlled clinical trial"[pt] OR randomized[tiab] OR placebo[tiab] OR "drug therapy"[sh] OR randomly[tiab] OR trial[tiab] OR groups[tiab]

#12 #3 AND #10 AND #11

**Search strategy for Embase**

#1 'alzheimer disease'/exp

#2 'dementia'/exp

#3 'alzheimer*’: ab,ti OR 'dement*':ab,ti

#4 #1 OR #2 OR #3

#5 'transcranial magnetic stimulation'/exp

#6 'transcranial magnetic stimulation*':ab,ti OR 'tms':ab,ti OR 'rtms':ab,ti

#7 #5 OR #6

#8 'Drug Therapy '/exp

#9 'Therapy, Drug':ab,ti OR 'Drug Therapies':ab,ti OR 'Therapies, Drug':ab,ti OR 'Chemotherapy':ab,ti OR 'Chemotherapies':ab,ti OR 'Pharmacotherapy':ab,ti OR 'Pharmacotherapies':ab,ti OR 'Inhibitors, Cholinesterase':ab,ti OR 'Anticholinesterase Drugs':ab,ti OR 'Drugs, Anticholinesterase':ab,ti OR 'Anticholinesterases':ab,ti OR 'Anticholinesterase Agents':ab,ti OR 'Agents, Anticholinesterase':ab,ti OR 'Anti Cholinesterases':ab,ti OR 'Anti Cholinesterases':ab,ti OR 'Cholinesterase Inhibitors':ab,ti OR 'Irreversible':ab,ti OR 'Inhibitors, Irreversible Cholinesterase':ab,ti OR 'Irreversible Cholinesterase Inhibitors':ab,ti OR'Cholinesterase Inhibitors, Reversible':ab,ti OR 'Inhibitors, Reversible Cholinesterase':ab,ti OR'Reversible Cholinesterase Inhibitors':ab,ti OR 'Acetylcholinesterase Inhibitors':ab,ti OR 'Inhibitors, Acetylcholinesterase':ab,ti OR 'Memantine':ab,ti OR 'donepezil':ab,ti OR'rivastigmine':ab,ti OR'galantamine':ab,ti #10 #8 OR #9

#11 'randomized controlled trial'/exp OR 'controlled clinical trial'/exp OR 'randomized':ti,ab OR 'placebo':ti,ab OR 'drug therapy':lnk OR 'randomly':ti,ab OR 'trial':ti,ab OR 'groups':ti,ab

#12 #4 AND #7 AND #10 AND #11

**Search strategy for Cochrane Library**

#1 MeSH descriptor: [Alzheimer Disease] explode all trees

#2 MeSH descriptor: [Dementia] explode all trees

#3 (alzheimer* or dement*): ti,ab,kw (Word variations have been searched)

#4 #1 or #2 or #3

#5 MeSH descriptor: [Transcranial magnetic stimulation] explode all trees

#6 Transcranial magnetic stimulation:ti,ab,kw (Word variations have been searched)

#7 #5 or #6

#8 MeSH descriptor: [Drug Therapy] explode all trees

#9 (Therapy, Drug OR Drug Therapies OR Therapies, Drug OR Chemotherapy OR

Chemotherapies OR Pharmacotherapy OR Pharmacotherapies OR Inhibitors, Cholinesterase OR Anticholinesterase Drugs OR Drugs, Anticholinesterase OR Anticholinesterases OR Anticholinesterase Agents OR Agents, Anticholinesterase OR Anti-Cholinesterases OR Anti Cholinesterases OR Cholinesterase Inhibitors, Irreversible OR Inhibitors, Irreversible Cholinesterase OR Irreversible Cholinesterase Inhibitors OR Cholinesterase Inhibitors, Reversible OR Inhibitors, Reversible Cholinesterase OR Reversible Cholinesterase Inhibitors OR Acetylcholinesterase Inhibitors OR Inhibitors, Acetylcholinesterase OR Memantine OR donepezil OR rivastigmine OR galantamine) :ti,ab,kw (Word variations have been searched)

#10 #8 or #9

#11 #4 and #7 and #10
