# Supplementary material for: Repetitive transcranial magnetic stimulation may be superior to drug therapy in the treatment of Alzheimer's disease: A systematic review and Bayesian network meta‐analysis
Source: CNS Neurosci Ther. 2023 Apr 23;29(10):2912–24. doi: 10.1111/cns.14228 (PMC10493651; doi:10.1111/cns.14228)
Supplement: Supplementary file 2 — Data S2: [file CNS-29-2912-s002.docx]

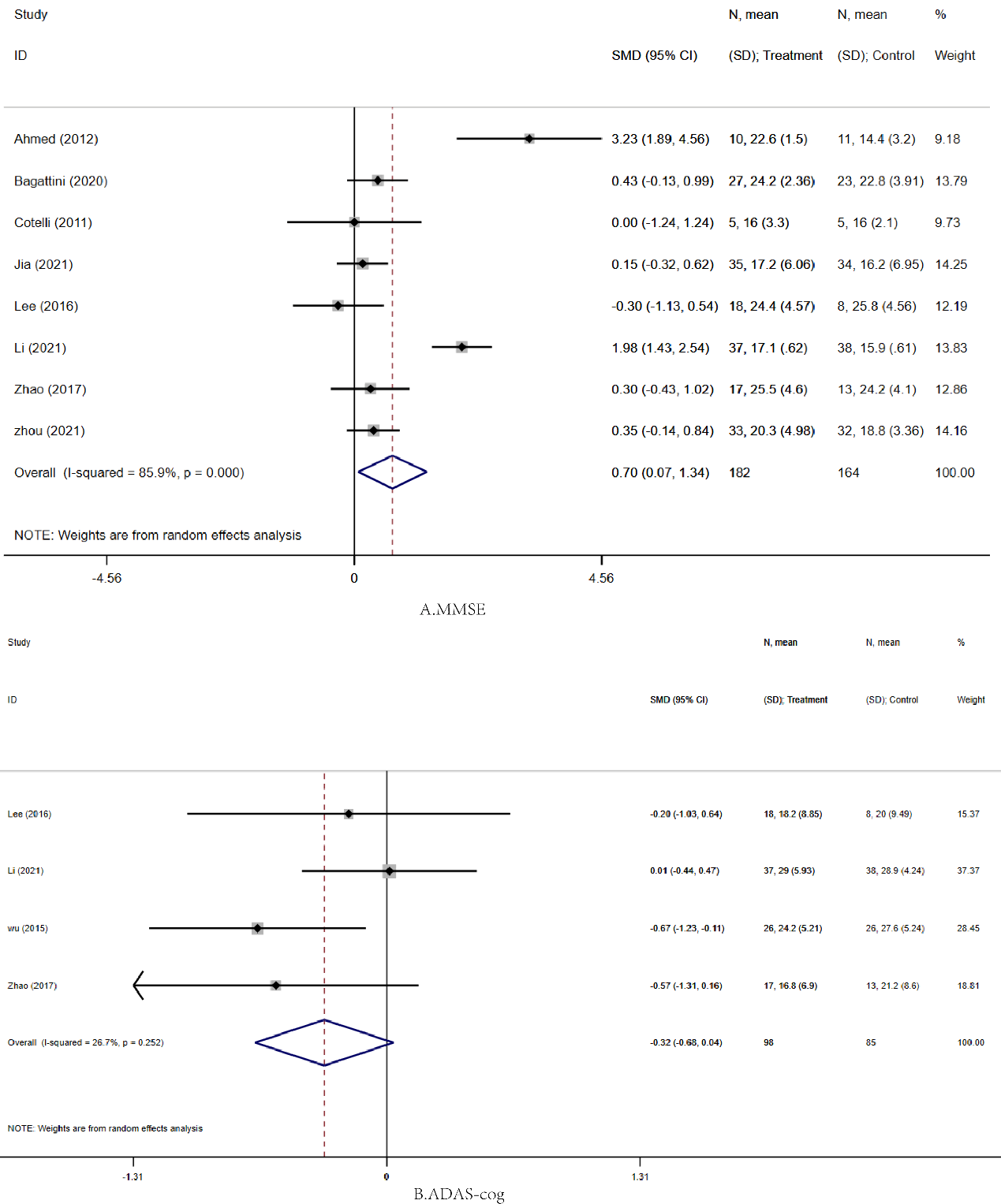


**Supplementary2 Meta-analysis of the effects of rTMS on global cognition in AD.**

**
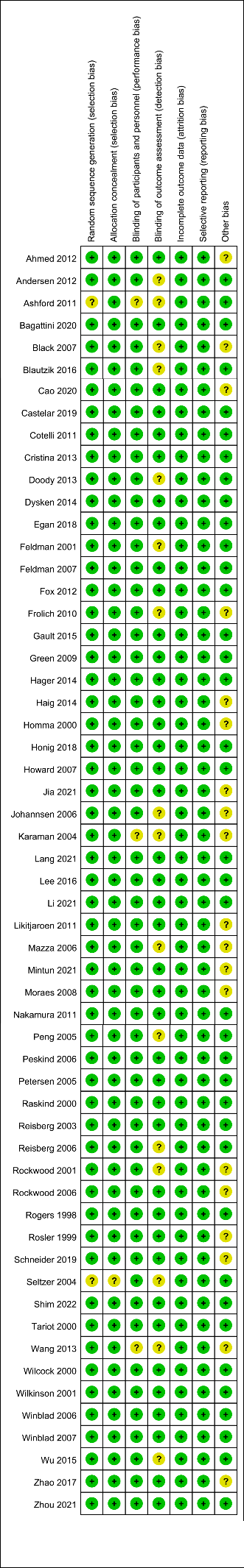
** **Supplementary3 Risk of bias summary**

**
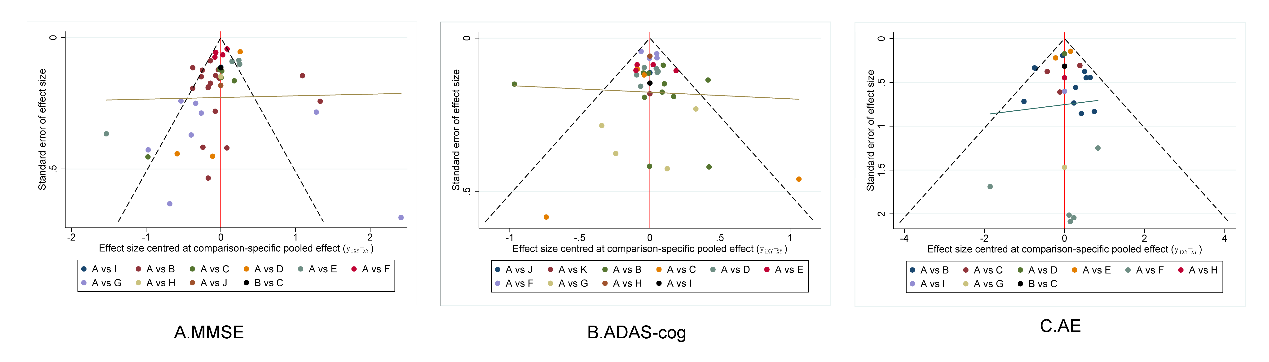
**

**Supplementary4 A comparison-adjusted funnel, [(A) MMSE results, (B) ADAS-cog scale, (C) AE, Adverse Events.**
